# Practice of immersion in hot water to relieve pain in neonatology: an integrative review

**DOI:** 10.1590/0034-7167-2023-0260

**Published:** 2024-02-26

**Authors:** Danton Matheus de Souza, Maria Eduarda Pires Yamamoto, Joese Aparecida Carvalho, Vanderlei Amadeu da Rocha, Vanessa Dias Fogaça, Lisabelle Mariano Rossato

**Affiliations:** I Universidade de São Paulo. São Paulo, São Paulo, Brazil; II Universidade de São Paulo, Hospital Universitário. São Paulo, São Paulo, Brazil

**Keywords:** Pain, Immersion, Water, Infant, Newborn, Nursing, Dolor, Inmersión, Agua, Recién Nacido, Enfermería, Dor, Imersão, Água, Recém-Nascido, Enfermagem

## Abstract

**Objectives:**

to identify immersion use in hot water to relieve pain in newborns.

**Methods:**

an integrative literature review, carried out in the PubMed, VHL, EMBASE, Scopus, CINAHL, Cochrane and SciELO databases, with investigations in English, Spanish, French or Portuguese, published between 2002 and 2022. The Health Sciences Descriptors (DeCS) and Medical Subject Headings (MeSH) were used to answer the following question: what are the uses of hot water immersion in relieving pain in newborns?

**Results:**

nine studies were included, mainly Brazilian, experimental, with a predominance of strong and moderate levels of evidence. Hydrotherapy and bath use (immersion and bandaging) was observed, promising interventions in reducing pain scores, assessed using scales, physiological and endocrine parameters.

**Conclusions:**

hot water proved to be a promising non-pharmacological intervention in relieving pain in infants in different contexts.

## RESUMO

**Objetivos:**

identificar o emprego da imersão em água quente no alívio da dor em neonatos.

**Métodos:**

revisão integrativa da literatura, realizada nas bases de dados PubMed, BVS, EMBASE, Scopus, CINAHL, Cochrane e SciELO, com investigações em inglês, espanhol, francês ou português, publicadas entre 2002 e 2022. Foram utilizados os Descritores em Ciências da Saúde (DeCS) e *Medical Subject Headings* (MeSH) para responder à seguinte inquietação: quais são os empregos da imersão em água quente no alívio da dor em neonatos?

**Resultados:**

foram incluídos nove estudos, principalmente brasileiros, experimentais, predominando o nível de evidência forte e moderada. Foram observados o emprego da hidroterapia e banhos (imersão e enfaixado), intervenções promissoras na redução do escore álgico, avaliados por meio de escalas, parâmetros fisiológicos e endócrinos.

**Conclusões:**

a água quente se mostrou uma intervenção não farmacológica promissora no alívio da dor de neonatos em diferentes contextos.

## RESUMEN

**Objetivos:**

identificar el uso de la inmersión en agua caliente para aliviar el dolor en recién nacidos.

**Métodos:**

revisión integrativa de la literatura, realizada en las bases de datos PubMed, BVS, EMBASE, Scopus, CINAHL, Cochrane y SciELO, con investigaciones en inglés, español, francés o portugués, publicadas entre 2002 y 2022. Se utilizaron los Descriptores de Ciencias de la Salud (DeCS) y los *Medical Subject Headings* (MeSH) para responder la siguiente pregunta: ¿cuáles son los usos de la inmersión en agua caliente para aliviar el dolor en los recién nacidos?

**Resultados:**

fueron incluidos nueve estudios, principalmente brasileños, experimentales, con predominio de niveles de evidencia fuerte y moderado. Se observó el uso de hidroterapia y baños (inmersión y vendaje), intervenciones prometedoras en la reducción de las puntuaciones de dolor, evaluadas mediante escalas y parámetros fisiológicos y endocrinos.

**Conclusiones:**

el agua caliente demostró ser una intervención no farmacológica prometedora para aliviar el dolor en neonatos en diferentes contextos.

## INTRODUCTION

Pain in neonatology is a frequent phenomenon. It is estimated that all newborns experience pain at some point in their early life, such as during vaccinations, with an increased likelihood of pain in cases of hospital admissions^(1)^. In Brazil, in a cross-sectional study carried out with 171 records of newborns (zero to 28 days of life corrected), assessed over the course of seven days of hospital admission in a Neonatal Intensive Care Unit (NICU), 102 records of pain in the hospital admission route, assessed by the Neonatal Infant Pain Scale (NIPS). 104 records were found in the nursing notes (crying, agitation and expression of pain) and also the submission of 4,765 painful procedures with an average of six per day and 27 per admission^(2)^.

Pain is defined as “an unpleasant sensory and emotional experience associated with actual or potential tissue damage, or described in terms of such damage”, with physical, emotional, social, and spiritual components^(3)^. The World Health Organization (WHO) recognizes that verbal reporting is only one of the components for its expression, as the inability to communicate does not invalidate the possibility of human beings feeling pain, as in the case of newborns^(1)^. However, despite progress on the topic, a Brazilian study carried out with 51 professionals from the nursing team indicated that 16% believed that newborns did not feel pain and 6% that experiencing pain repeatedly does not have long-term consequences^(4)^.

In view of pain relief, the WHO recommends a comprehensive approach to treatment as a principle, with a combination of pharmacological and non-pharmacological interventions, individualized to pain complaint^(1)^. In newborns, a systematic review of non-pharmacological interventions indicated the possibility of using sweetened solutions, non-nutritive sucking, breastfeeding and sensory stimulation (holding, skin care, kangaroo care), which showed effectiveness in reducing behavioral and physiological responses to pain^(5)^. However, in a Brazilian investigation, carried out with 90 newborns in the NICU, followed for three days, it indicated that, of 2,732 invasive procedures, only 540 non-pharmacological interventions were used (e.g., light reduction) and 216 pharmacological interventions (e.g., continuous opioid)^(6)^.

There is a need for advancement in the study of the topic, and nurses stand out in this journey as an essential agent in pain management, especially with their autonomy in non-pharmacological intervention use^(1)^. However, a systematic literature review indicated that, although pain relief is an essential competence of neonatal nurses, they devalue pain, mainly due to unfavorable beliefs, as in the case of non-pharmacological intervention use^(7)^. In this context, scientific research proves to be an essential ally, being a starting point for transforming this scenario^(3,8)^.

Specifically, here the authors will work on immersion use in hot water as a promising non-pharmacological intervention for pain relief, considering immersion with a water temperature between 36 and 39°C as hot^(9-10)^. Water use has occurred since the time of Hippocrates (460-375 BC), with widespread use throughout Greece and ancient Rome and the construction of numerous therapeutic spaces, as a source of curing illnesses. However, over the years, its use was reduced by intense scientific growth, mainly pharmacological interventions^(11-13)^. However, with the resurgence of emphasis on non-pharmacological interventions, it is necessary to look again at water use.

Hot water can be considered a non-pharmacological intervention due to its physical properties (hydrostatic pressure, buoyancy, turbulence and temperature), which act on nociception, with blood vasodilation that leads to dissipation of inflammatory cells, an increase in cenesthetic stimuli in the skin and blood flow and a reduction in the sympathetic system’s activity, thus promoting relaxation and pain relief^(9,11)^. It is worth noting that, in infants, water can have subjective effects, due to the reminder of the intrauterine environment, leading to a feeling of security^(9,14)^.

There are different ways of using hot water for therapeutic purposes which, despite having different purposes, make use of the previous properties in a common way, such as bathing, *ofurô* or tummy bath (bucket), spa therapy, hydrotherapy (motor physiotherapy in an aqueous environment) and watsu therapy (physiotherapy in an aqueous environment, using buoyancy)^(10,15-16)^. However, when portraying infants with pain, no literature review was found on the subject, making it necessary to carry out a synthesis to be able to identify intervention use and gaps in the studies, enabling using the intervention safely and the advancement of scientific literature.

## OBJECTIVES

To identify immersion use in hot water to relieve pain in newborns.

## METHODS

This is an integrative literature review. This methodology was chosen in order to build a synthesis of the phenomenon and because using hot water is linked to different interventions, and it is not possible to systematize the data at this time, with this study being the starting point in the line of investigation into thermotherapy. To this end, five steps were taken: theme identification and hypothesis selection; establishment of eligibility criteria and literature search; definition of the information to be extracted; assessment of included studies; and interpretation/presentation of results^(17)^.

The review was guided by the following research question: what are the uses of hot water immersion in relieving pain in newborns? It was built based on the PICo mnemonic: P (population): infants (considered from zero to 28 days of corrected age)^(18)^; I (intervention): immersion in hot water as a pain relief intervention; Co (context): scientific literature. It is reiterated that, initially, the review was formulated to address infants, children and adolescents; however, with article assessment, a predominance of infants and few studies on other profiles was noted. Aiming to reduce the heterogeneity of the population included in the investigation, it was decided to standardize studies only with newborns.

Based on the question, the following descriptors were chosen: P: Child; Infant; Newborn; Child, Preschool; Preschool child; Toddler; Pre-School e All Child; I: Baths; Hydrotherapy; *Ofurô*; Immersion; Hot tub; Bathing; Bathing and Baths; Spa therapy; Water e Hot water; O: Pain; Analgesia; Pain Management; Pain relief; Pain reduction and Pain Control. The descriptors were linked with the Boolean operators AND or OR. The PubMed, VHL, EMBASE, Scopus, CINAHL, Cochrane and SciELO databases were used. For each database, a search strategy was formulated, with the help of a librarian specializing in integrative review, in December 2022.

Original articles, which portray an intervention that used immersion in hot water (temperature between 36-39°C)^(9-10)^ in infants (zero to 28 days of corrected age)^(18)^, with pain assessed by a validated scale, quantitative approach, language in Portuguese, English, Spanish or French, published between January 2002 and December 2022, were included. Unavailable articles, opinion articles, expert consensus, use of experimental models, research protocols, abstracts, editorials and theses/dissertations were excluded.

For data collection, the following steps were followed: reading titles and abstracts; reading the article in full; search for evidence based on article references; and data collection. It is worth noting that all these steps were carried out in pairs of researchers, independently. In case of disagreement, a third researcher participated in the stage and made the final decision.

The data were organized in a Microsoft Excel^®^ spreadsheet, with the following variables: authors; year of publication; country; objective; method; assessment of level of evidence; sample size; characterization of participants; data collection location; type, time and temperature of water during intervention; association of immersion in water with another intervention; main results; and conclusion. Assessment of the level of evidence based on the JBI classification includes: level I: systematic review or meta-analysis; level II: randomized controlled clinical trial; level III: controlled clinical trial without randomization/quasi-experimental studies; level IV: well-designed cohort or case-control studies; level V: systematic review of qualitative and descriptive studies; level VI: descriptive or qualitative studies; and level VII: opinion of authorities or report of experts. Levels are classified as strong (I and II), moderate (III to V) and weak (VI to VII)^(19)^.

As this was a literature review, study submission and ethical assessment was not necessary.

## RESULTS

**Figure 1:**
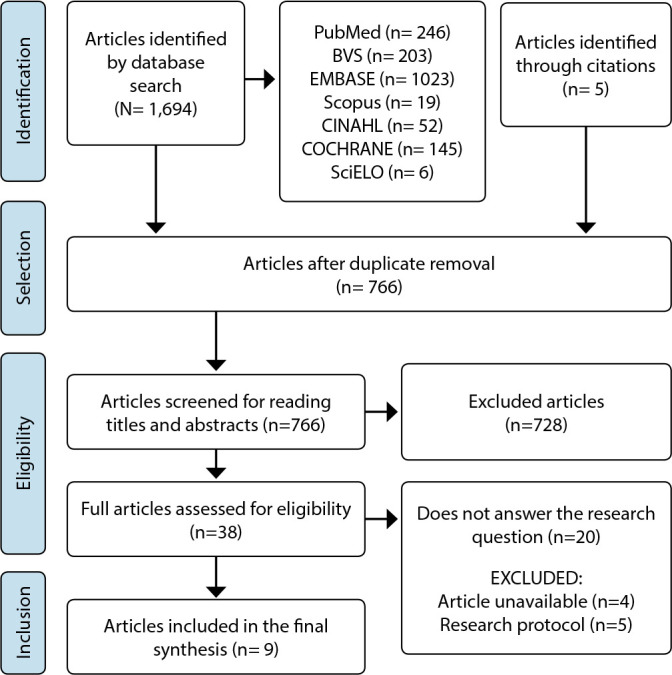
PRISMA flowchart: search and selection of included articles, São Paulo, São Paulo, Brazil, 2023

Preliminarily, 766 studies were found and, after critical assessment, nine studies were selected for the final synthesis (Figure 1).

There was a prevalence of articles from the last 5 years, with six Brazilian and three Turkish publications, with strong and moderate levels of evidence, with newborns, admitted to the NICU, assessed for pain using validated scales. In addition to the scales, all articles used at least one vital sign, such as heart rate (HR), respiratory rate (RR) or mean arterial pressure (MAP), and one study used salivary cortisol (endocrine assessment parameter). A single exposure to the investigated intervention prevailed, with an immersion time between two and 10 minutes. The interventions reported were hydrotherapy^(8-10,20-21)^, sponge bath and immersion (with the child bandaged or not swaddled)^(16,22)^, immersion bath^(23)^ and an intervention called “first bath”^(14)^.

All studies that used hydrotherapy indicated a time of 10 minutes, with one session. In one of the studies^(8)^, pain relief was not indicated, but there were changes in physiological parameters. Studies that compared sponge baths with swaddled immersion^(16)^ or immersion alone^(22)^ with newborns in NICUs demonstrated lower pain scores and changes in physiological parameters. Immersion baths were assessed in a study^(23)^ with newborns in NICUs, demonstrating pain relief. Study called its intervention “first bath”, and presented the following characteristics: systematization of the immersion process in a bathtub; prior feeding and hygiene of the newborn; immersion with the wrapping technique and controlled temperature; carried out by nurses and later by their technical team, with a greater impact on stress and pain scores, compared to standard bath^(14)^.

**Figure 2:**
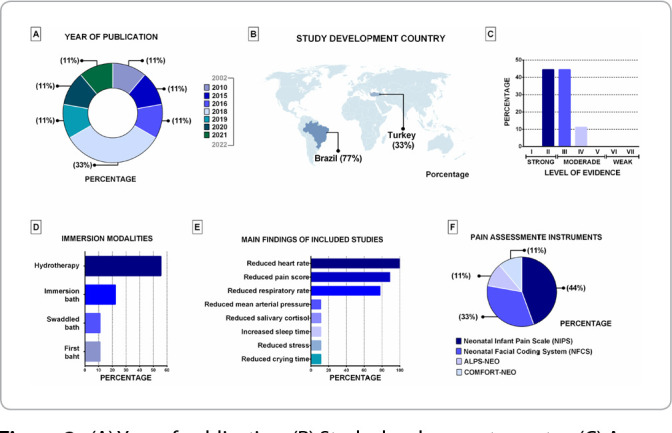
(A) Year of publication; (B) Study development country; (C) Assessment of level of evidence; (D) Characterization of immersion modalities; (E) Main findings of included studies; (F) Characterization of pain assessment instruments, São Paulo, São Paulo, Brazil, 2023

In summary, the interventions provided effects in relieving acute pain^(8-10,14,16,20-23)^, observed in 90% of articles^(9-10,14,16,20-23)^. Only one investigation reported no improvement in pain, but a reduction in physiological and endocrine parameters^(8)^. Furthermore, there was a reduction in HR^(8-10,14,16,20-23)^, RR^(8,10,14,16,20-21,23)^ and MAP^(21)^. Other parameters were reported, such as increased sleep time^(20)^, reduced salivary cortisol^(8)^, crying time and stress score^(16)^ (Chart 1 and Figure 2).

**Chart 1:** Characterization of hot water immersion use as an intervention for pain relief, São Paulo, São Paulo, Brazil, 2023

Author Year Country	Objective	Design Level of evidence	Participants Study site Pain scale	Type of immersion, time and water temperature during intervention	Results Conclusion
**INTERVENTION: HYDROTHERAPY**					
Vignochi C et al.; 2010; Brazil^(20)^	Assess the effects of hydrotherapy on improving sleep quality and reducing pain in PTNB.	Uncontrolled clinical trial; Level III	12 PTNB; NICU; NFCS + physiological parameters	Hydrotherapy; 10 minutes; 37°C	Lower pain scores, HR, RR and longer sleep time.
Barbosa LPC et al.; 2015; Brazil^(10)^	Assess the impact of hydrotherapy on newborns admitted to hospital.	Quasi-experimental study; Level III	10 newborns >72 hours old; NICU; NIPS + physiological parameters	Hydrotherapy; 10 minutes; 37 and 38°C	Greater reduction in pain score, RR and HR.
Tobinaga WCO et al.; 2016; Brazil^(8)^	Investigate the effects of hydrotherapy on salivary cortisol, hemodynamics and pain level in PTNB.	Quasi-experimental study; Level III	15 PTNB; NICU; NIPS + physiological parameters + endocrine parameters	1 hydrotherapy session; 10 minutes; 37°C	There were no significant changes in the pain score, but there was a reduction in HR, RR and salivary cortisol level
Novakoski KRM et al.; 2018; Brazil^(9)^	Analyze the effects of hydrotherapy on pain and physiological variables in clinically stable PTNB.	Quasi-experimental study; Level III	22 PTNB; NICU; NFCS + physiological parameters	Hydrotherapy; 10 minutes; 36 and 37.5°C	Lower pain score immediately and after 10 minutes and lower HR.
Cecconello BW et al; 2021; Brazil^(21)^	Assess the effect of hydrotherapy on pain and vital signs of PTNB in the ICU.	Observational study; Level VI	54 medical records of PTNB; NICU; NFCS + physiological parameters	Hydrotherapy; 10 minutes; 36.5 and 37.5°C	Significant reduction in pain score, HR, RR and MAP.
**INTERVENTIONS: IMMERSION BATH, SWADDLED OR “FIRST BATH”**					
Ceylan SS, et al.; 2018; Turkey^(16)^	Determine the effects of sponge baths and swaddle baths on pain, crying time, and physiological variables.	Randomized clinical trial; Level II	35 PTNB; NICU; ALPS-Neo + physiological parameters	Immersion bath with swaddling; Between 3.8 ± 0.6 minutes; 37.5±0.8°C	Lower pain score, HR, RR, crying time and stress score in swaddled bath.
Gunay U, et al; 2018; Turkey^(23)^	Assess the difference between the pain of newborns who took a bath and those who did not.	Randomized clinical trial; Level II	70 newborns >28 weeks, with moderate or severe pain; 35 in the CG and IG; NICU; NIPS + physiological parameters	Immersion bath compared to no intervention; Between 2-3 minutes; 37 and 38°C	Greater reduction in pain score, HR and RR in the immersion bath.
Tasdemir HI, et al. 2019; Turkey^(22)^	Assess the effectiveness of bath and sponge baths on the physiological parameters and comfort of late PTNB.	Randomized clinical trial; Level II	120 PTNB (34-36 weeks); NICU; COMFORTneo + physiological parameters scale	(Immersion) bath Average of 3.74 minutes; 37 and 38°C	Significant reduction in pain score and HR in immersion bath.
Lima RO, et al.; 2020; Brazil^(14)^	Compare the nursing intervention “First bath” with the “Institutional standard operating procedure (SOP) bath” on neonatal behavior.	Randomized controlled clinical trial; Level II	33 TNB, with 15 in the CG and 18 in the IG; Rooming-in; NIPS + physiological parameters	Infant’s first bath versus standard operating procedure bath; 14 minutes; 38°C	Lower pain score, HR and RR after the first bath intervention.

*NB - newborn; PTNB - preterm newborn (<37 weeks); TNB - term newborn (>37 weeks); NICU - Neonatal Intensive Care Unit; CG - control group; IG - intervention group; HR - heart rate; RR - respiratory rate; MAP - mean arterial pressure; NIPS - Neonatal Infant Pain Scale; NFCS - Neonatal Facial Coding System; ALPS-Neo - Neonatal Pain and Stress Assessment Scale.*

## DISCUSSION

In this review, studies that assessed pain using validated scales were included, ensuring the quality standard for measuring the outcome. Furthermore, physiological and endocrine parameters were reported, enriching the results. Water temperature assessment is vitally important for ensuring the safety of the intervention. It is reiterated that harmful pain receptors are activated with exposure of the skin to water temperatures above 42°C^(24)^, therefore, control of hot water at 36-39°C is necessary.

In the face of pain, it is noted that there was a prevalence of assessment during some stressful or painful procedure, with a gap in the effect of immersion in hot water on acute pain associated with pathologies, such as respiratory problems. Interventions make use of water temperature that, when elevated, can act with nociceptors, reducing pain sensation^(3)^. However, future investigations with newborns with pain associated with the pathology are necessary to increase the range of use of interventions in clinical practice.

Immersion in water can interfere with infants’ extrauterine adaptation, with changes in thermal protection, increased oxygen consumption and changes in physiological parameters^(14,16)^, aspects inversely proportional to age, with preterm newborns being more vulnerable. In view of this aspect, there has been an increase in studies on bathing, thermal control and stress in newborns^(14)^. Since 1999, investigations have indicated the effectiveness of immersion in hot water at minimal stress, to the detriment of other modalities^(22)^, corroborating this review. However, this intervention has not yet been incorporated into the clinical practice of many institutions, requiring investment in implementation research.

Two clinical trials regarding water immersion use in infants were included in this review^(22-23)^. In one of these^(23)^, the intervention group received an immersion bath, while the control group did not receive any type of intervention or stressful stimulus, with measurement of pain and physiological parameters at the same time that one was taking a shower and the other was not receiving intervention. In the end, the study demonstrated less pain relief in infants who took a bath, however this allocation appears to be limiting and a possible confounding variable for the outcome, since bathing in infants, in itself, promotes stress, and when comparing a stressed infant with another without any stimulus, outcome measurement will be biased. When related to another study^(22)^, which compared the immersion bath with a sponge bath, the measurement bias appears to be reduced, since both groups were manipulated during the study.

Aiming to reduce stress, the literature continues to advance in the improvement of techniques for bathing newborns, such as “first bath” and “swaddled bath”.

The “first bath” intervention is systematically organized and carried out by nurses; however, evidence was conducted with term newborns that, unlike preterm newborns, already has greater physiological regulation to stressful procedures. The intervention application in clinical practice is questioned, considering that nurses are the agents indicated for the first implementation, but this professional is overloaded with demands in the health service^(3)^, having a technical team that works with a focus on assistance, carrying out the intervention for years with similar results^(14,16)^. It is worth considering whether it is worth standardizing a professional or investing in training with other team members.

As another possibility for stress relief, the swaddled bath emerged, which simulates the intrauterine environment and leads to promising effects on pain relief^(16,25)^. In this review, only one investigation involved swaddled bath, which was compared with sponge bath, being more effective in pain relief^(16)^. However, studies are needed to compare immersion bath and swaddled bath, since the second uses two interventions, raising doubts as to whether pain relief occurs through immersion or facilitated restraint. The Ministry of Health^(25)^ already recommends using swaddled bath, but, similar to immersion bath, in preterm newborns, this method is rarely used.

Bathing, unlike hydrotherapy, is an intervention that can be used to relieve pain at home and in hospital settings by nurses. In children, due to their physiological organization, bathing is no longer stressful and becomes a moment of relaxation, however this review only worked with infants (term newborns or preterm newborns), and studies that work with different age groups are essential, with the redefinition of a common hygiene and relaxation intervention to a non-pharmacological intervention disseminated in different contexts.

Although this review only indicates immersion in hot water, it is worth reflecting, in future studies on sprinkling, on the technique performed by family members at home with infants, in which bathing is done together (child + family), but without investigations into the clinical repercussions, and in children over three years old, there is daily use of the intervention, being more accessible than the immersion method.

Another intervention that uses immersion in hot water is hydrotherapy, recommended for motor and respiratory physiotherapy in infants^(8-10)^. It is worth noting that physiotherapists are the ones who work with the technique, however, although nurses have a key role in pain relief, other professionals must be empowered with techniques that help their clinical practice in stressful procedures.

Hydrotherapy has an impact on quality of life, with impacts on the motor, cardiac, respiratory system and mental health^(10,21)^. A study with hydrotherapy with preterm newborns did not indicate pain relief, but reduced physiological parameters (HR and RR) and endocrine parameters (salivary cortisol)^(8)^, parameters that can be considered indicators of pain relief in newborns^(3)^. Furthermore, although pain scales are psychometrically validated, they depend on a subjective assessment by health professionals, which can impact outcome measurement, especially when there is no blinding.

In this review, studies related to bathing were randomized clinical trials, different from hydrotherapy, with only one clinical trial^(20)^, quasi-experimental studies^(8-10)^ and one observational study prevailing^(21)^. The difference in design proves to be a limiting factor for assessing the intervention, since quasi-experimental studies do not have a control group, which limits us to saying whether the intervention actually promotes enhancing effects in relation to what is already done in clinical practice. Furthermore, observational studies are limited by their only measurement and without the possibility of associating them with causality. Therefore, results involving hydrotherapy should be viewed with caution.

In addition to pain, a study with hydrotherapy reported an increase in sleep time^(20)^, but the measurement of this variable is questionable, since sleep can be influenced by numerous determinants, even more so in a hospital admission, such as manipulation, light and sound. To this end, longitudinal monitoring is necessary, in addition to environmental control and more exposure to the intervention to reach a reliable report of this variable.

Another study with hydrotherapy reported no pain relief, but modified physiological and endocrine parameters^(8)^. It is understood that the relatively small sample size (15 participants) as well as the complexity of neonatal pain assessment may have made the association of hydrotherapy with pain scores unfeasible. Still, it is possible to infer pain relief from the reduction in cortisol levels after hydrotherapy and highlight the importance of exploring salivary biomarkers as complementary measures in pain assessment, especially in this vulnerable population exposed to a high number of potentially painful procedures. Furthermore, the purpose of hydrotherapy is physical therapy, and the secondary outcome is pain relief. There is a need for multiple sessions^(8-10,15,20-21)^. Therefore, longitudinal investigations are needed to identify whether everyday exposure to water will maintain its effects on pain relief.

Another type of water immersion is tummy bath, which was not covered in the studies in this review, but is an approach that has gained visibility in the area of neonatology. Tummy bath consists of immersing the child in a bucket of hot water, vertically, up to the height of the clavicle, aiming for relaxation, and is recommended by the Ministry of Health^(25)^. Its use consists of gentle mobilizations in an assisted float^(25-26)^. In one of the included studies^(8)^, hydrotherapy was performed with the child in a bucket. The technique may resemble tummy bath^(26)^, but it should not be confused, as its purpose consists of motor physiotherapy with immersion in bathtubs, buckets or swimming pools, while the tummy bath is not linked to physical exercise. Therefore, the techniques must be properly named.

In Brazil, researchers translate keywords, such as “Hot tub” and “Tub bath”, to *ofurô*, however there is a conceptual error that results in biased investigations. The term colloquially used outside the country is “Tummy bath”, however, there is a lack of investigations into the effectiveness of the intervention, despite nurses having an active role in its application, making it necessary to conduct experimental studies.

For future studies, it is suggested to carry out systematic reviews with meta-analysis on each isolated modality, such as clinical trials with blinding of evaluators, qualitative investigations, pain assessment performed by the sum of scales, physiological and endocrine parameters, association of already effective non-pharmacological interventions, such as music, with immersion in randomized studies, and nurses as agents for building future evidence, empowering the science of the profession.

### Study limitations

This review has the following limitations: the inclusion of different immersion modalities, which implies a careful look at the results; studies addressing pain relief as a secondary outcome to physical manipulation or in investigations focusing on assessing infants’ body temperature; lack of mention of variables, such as using analgesics concomitantly with the intervention; citation from the carrying out agent (mother, unit professional or researcher) of the intervention, as this could influence the outcome.

### Contributions to nursing

It is hoped that this review can demonstrate to nurses, who work with infants, the potential of immersion in hot water as a non-pharmacological intervention for pain relief, moving forward in order to equip professionals in the face of an intervention that can be carried out in clinical practice, with available materials, making pain important, understood, visible and better managed.

## CONCLUSIONS

This review demonstrated promising effects of using hot water immersion in relieving pain in infants. The modalities of hydrotherapy and baths (immersion and swaddled) were cited in experimental studies, with strong and moderate levels of evidence, built in Brazil, demonstrating the pioneering spirit of national science in the study of the phenomenon.
